# *Legionella* hijacks the host Golgi-to-ER retrograde pathway for the association of *Legionella-*containing vacuole with the ER

**DOI:** 10.1371/journal.ppat.1009437

**Published:** 2021-03-24

**Authors:** Mio Kawabata, Honoka Matsuo, Takumi Koito, Misaki Murata, Tomoko Kubori, Hiroki Nagai, Mitsuo Tagaya, Kohei Arasaki

**Affiliations:** 1 School of Life Sciences, Tokyo University of Pharmacy and Life Sciences, Hachioji, Tokyo, Japan; 2 Department of Microbiology, Graduate School of Medicine, Gifu University, Gifu, Japan; 3 G-CHAIN, Gifu University, Gifu, Japan; University of Oxford, UNITED KINGDOM

## Abstract

*Legionella pneumophila* (*L*. *pneumophila*) is a gram-negative bacterium that replicates in a compartment that resembles the host endoplasmic reticulum (ER). To create its replicative niche, *L*. *pneumophila* manipulates host membrane traffic and fusion machineries. Bacterial proteins called *Legionella* effectors are translocated into the host cytosol and play a crucial role in these processes. In an early stage of infection, *Legionella* subverts ER-derived vesicles (ERDVs) by manipulating GTPase Rab1 to facilitate remodeling of the *Legionella*-containing vacuole (LCV). Subsequently, the LCV associates with the ER in a mechanism that remains elusive. In this study, we show that *L*. *pneumophila* recruits GTPases Rab33B and Rab6A, which regulate vesicle trafficking from the Golgi to the ER, to the LCV to promote the association of LCV with the ER. We found that recruitment of Rab6A to the LCV depends on Rab33B. *Legionella* effector SidE family proteins, which phosphoribosyl-ubiquitinate Rab33B, were found to be necessary for the recruitment of Rab33B to the LCV. Immunoprecipitation experiments revealed that *L*. *pneumophila* facilitates the interaction of Rab6 with ER-resident SNAREs comprising syntaxin 18, p31, and BNIP1, but not tethering factors including NAG, RINT-1, and ZW10, which are normally required for syntaxin 18-mediated fusion of Golgi-derived vesicles with the ER. Our results identified a Rab33B-Rab6A cascade on the LCV and the interaction of Rab6 with ER-resident SNARE proteins for the association of LCV with the ER and disclosed the unidentified physiological role of SidE family proteins.

## Author summary

*Legionella pneumophila* causes a sever pneumonia called Legionnaires’ disease and a threat of this disease has increased on a world-wide scale. As a feature of *L*. *pneumophila*, it secrets over 300 bacterial effectors to adapt and survive inside the host and many of effectors modify the host proteins in a unique manner. *L*. *pneumophila* is known to travel inside the host and final destination of this pathogens is the host ER. In the initial step of this travel, *L*. *pneumophila* subverts host early vesicular trafficking to remodel the membrane composition of *Legionella*-containing vacuole (LCV). Although this remodeling process has been well characterized, the molecular machinery of association of remodeled vacuoles with the ER is still obscure. This paper shows that the host GTPases Rab6A and Rab33B, both of which control Golgi-to-ER traffic, are recruited to the LCV in a cascade manner and are required for the association of LCVs with the ER through the interaction between Rab6A and ER-resident t-SNARE proteins. Of note, we demonstrate that a bacteria-specific Rab33B modification called phosphoribosyl-ubiquitination by *Legionella* effectors proteins of the SidE family is essential for the recruitment of Rab33B to the LCV.

## Introduction

Most intracellular pathogens modulate the host membrane transport and fusion systems to survive and replicate inside the host. To better understand how intracellular pathogens control host trafficking and fusion machineries, we have been studying the intracellular survival strategy of *Legionella pneumophila*. *L*. *pneumophila* is an intracellular pathogen that subverts host membrane traffic [1,2]. Human infection with *L*. *pneumophila* may occur as an incidental exposure to the pathogen followed by phagocytic uptake of the organism into macrophages in the human lung. After entry into host cells, *L*. *pneumophila* evades delivery to lysosomal compartments [3]. Furthermore, *L*. *pneumophila* co-opts small vesicles that bud from the host endoplasmic reticulum (ER), and the *Legionella*-containing vacuole (LCV) membrane is converted into the ER-Golgi intermediate compartment (ERGIC)-like structure [4] through a process called “remodeling”. In the remodeling, *L*. *pneumophila* manipulates host GTPase Rab1, which is a tether between ER-derivded vesicles (ERDVs) and ERGIC/Golgi membranes [5]. Furthermore, *L*. *pneumophila* promotes the association of Sec22b, an *N*-ethylmaleimide-sensitive factor (NSF) attachment protein receptor (v-SNARE) on the ERDV membrane, with syntaxin (Stx) 2, 3, 4 and SNAP23, plasma membrane located t-SNAREs, for efficient fusion of ERDVs with the LCV [6]. This remodeling is prerequisite for the association of the LCV with the host ER.

Of note, these processes are mediated by bacterial proteins called *Legionella* effectors, which are secreted into the host cytosol through the type IV secretion apparatus (also known as Dot/Icm apparatus) [1,7–10]. The molecular mechanism of the remodeling of the LCV has been well studied, and the *Legionella* effector DrrA (also called SidM) plays a central role in this remodeling: DrrA recruits Rab1 to the LCV and activates Rab1 through its guanine nucleotide exchanging factor (GEF) activity [11,12]. Furthermore, DrrA conjugates AMP to Rab1 via adenylyltransferase activity [13], and this conjugation prolongs the GTP state of Rab1 on the LCV [14]. Finally, DrrA facilitates non-canonical SNARE-mediated membrane fusion of ERDVs with the LCV [15]. A recent study demonstrated that some, but not all, components of the exocyst complex that functions as a tether between secretory vesicles and the plasma membrane in the host are recruited to the LCV by DrrA-activated Rab1 and subsequently facilitate the linkage of ERDVs with the LCV [16]. Although reticulon 4 and atlastin, which regulate the formation and stabilization of ER tubules, are localized to the LCV and are required for *Legionella* growth [17,18], the details of the association of the LCV with the ER after remodeling are not fully understood.

We here show that *L*. *pneumophila* utilizes a Rab33B-Rab6A cascade on the LCV for its association with the ER. Furthermore, Rab33B-dependent Rab6A distribution on the LCV facilitates the acquisition of LCV into the ER though the interaction of Rab6A with ER-resident SNARE proteins including syntaxin 18 (Stx18). Interestingly, the host Stx18-associated tethering complex (NRZ complex; [19]) neither participates in the Rab6A-ER-resident SNARE complex nor is recruited to the LCV. Of note, the recruitment of Rab33B to the LCV requires its phosphoribosyl-ubiquitination through SidE family effectors [20].

## Results

### Requirement of Rab6A and Rab33B for the Association of the LCV with the ER

**Fig 1:**
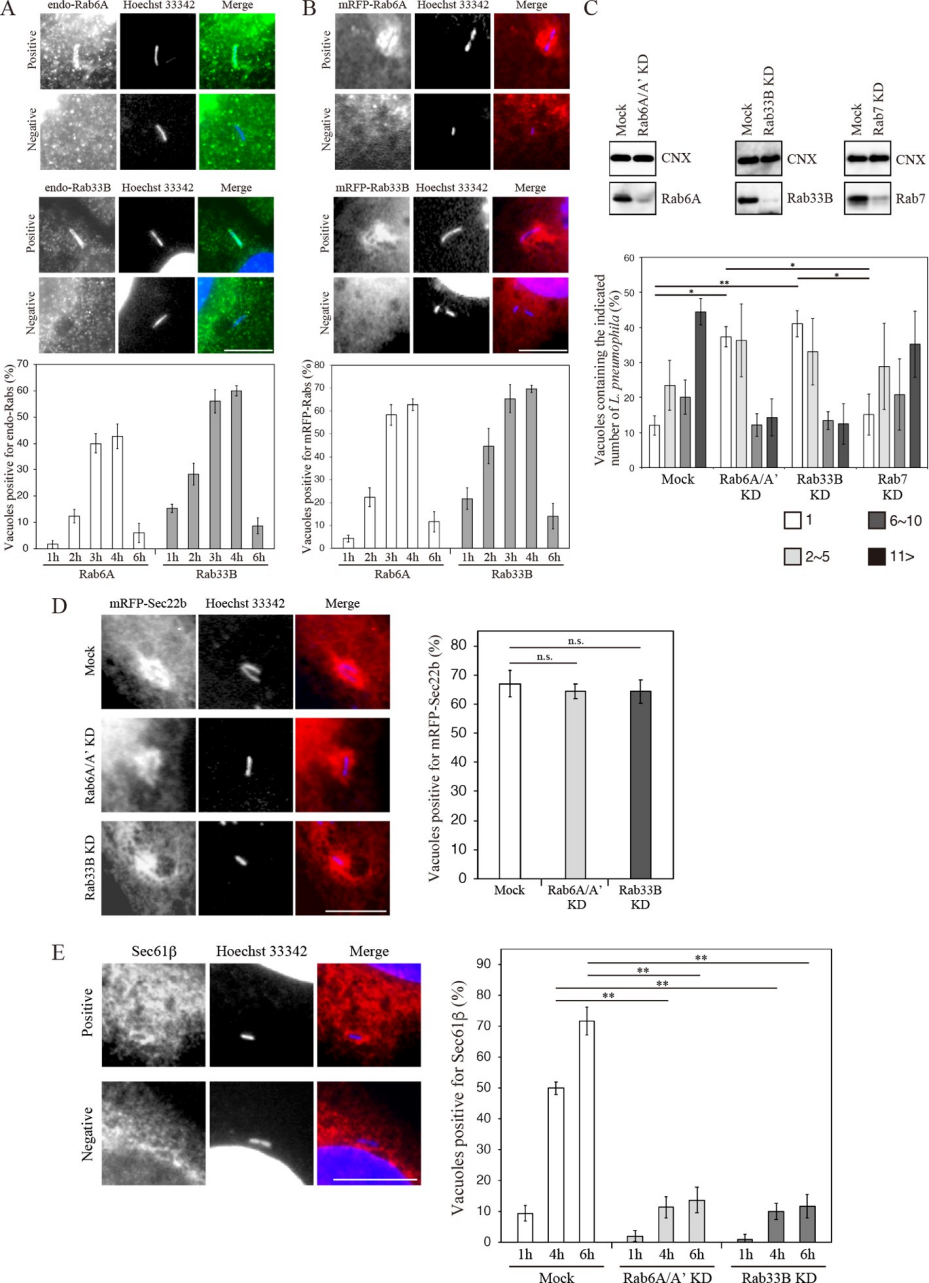
*L*. *pneumophila* subverts Rab6A and Rab33B function for the association of the LCV with the ER. (A and B) HeLa-FcγRII cells (A) or HeLa-FcγRII cells transfected with a plasmid for mRFP-Rab6A or -Rab33B for 24 h (B) were infected with *L*. *pneumophila* for the indicate times at MOI 10. After infection, cells were fixed and stained with an anti-Rab6A or -Rab33B antibody and Hoechst 33342 (A) or with Hoechst 33342 alone (B). Images show that typical vacuoles positive and negative for endogenous and expressed Rab6A (top and second rows) and Rab33B (third and bottom rows). Bar, 5 μm. The graphs show the percentage of vacuoles positive for endogenous or expressed Rab proteins. Values are the mean ± SD (n = 3, 100 vacuoles were scored in each experiment). (C) HeLa-FcγRII cells were transfected without (mock) or with siRNA targeting Rab6A/A’, Rab33B, or Rab7. At 72 h after transfection, the efficiency of siRNAs was assessed by the indicated antibodies. Mock and siRNA transfected HeLa-FcγRII cells were infected with *L*. *pneumophila* for 8 h at MOI 10. Intracellular growth of *L*. *pneumophila* was scored by counting bacteria residing in a single vacuole. The graph shows the percentage of vacuoles containing 1 bacterium (white bars), 2–5 bacteria (light gray bars), 6–10 bacteria (dark gray bars), and >11 bacteria (black bars). Values are the mean ± SD (*n* = 3, 100 vacuoles were scored in each experiment). *P < 0.05, **P < 0.01 (Tukey’s test). (D) HeLa-FcγRII cells were transfected without (top row) or with siRNA targeting Rab6A/A’ (middle row) or Rab33B (bottom row). At 48 h after transfection, the cells were additionally transfected with a plasmid for mRFP-Sec22b for 24 h, infected with *L*. *pneumophila* for 1 h, fixed, and stained with Hoechst 33342. Bar, 5 μm. The graph shows the percentage of vacuoles positive for mRFP-Sec22b. Values are the mean ± SD (n = 3, 100 vacuoles are scored in each experiment). n.s., not significant (Tukey’s test). (E) Mock and siRNA transfected HeLa-FcγRII cells were infected with *L*. *pneumophila* for the indicated times, fixed, and stained with an anti-Sec61β antibody and Hoechst 33342. Images show that typical vacuoles positive (top row) or negative (bottom row) for Sec61β. Bar, 5 μm. The graph shows the percentage of vacuoles positive for Sec61β at the indicate times. Values are the mean ± SD (*n* = 3, 100 vacuoles were examined in each experiment). **P < 0.01 (Tukey’s test).

Because Rab proteins play a pivotal role in the intracellular delivery of cargo molecules [21] and several Rabs are utilized by *L*. *pneumophila* [22], we speculated that some Rab protein(s) participates in LCV association with the ER. We therefore focused on Rab6A and Rab33B, both of which are known to be implicated in membrane traffic toward to the ER [23–25]. To assess whether Rab6A and Rab33B are implicated in *L*. *pneumophila* infection, we first examined the distribution of these Rabs on the LCV. As shown in Fig 1A and 1B, both endogenous and mRFP-tagged Rab6A and Rab33B were detected in a significant fraction of LCVs, and the recruitment of these Rabs to the LCVs are increasing at 2 h post infection, corresponding to the time when ER protein calnexin starts to accumulate on the LCV [26,27]. The fact that time course of the recruitment of mRFP-Rabs to the LCV is indistinguishable from that of endogenous Rabs recruitment, suggesting that the expression of mRFP-Rabs does not affect the *L*. *pneumophila* infection process in host cells. Importantly, a similar recruitment kinetics for these Rabs was observed in J774.1 macrophage cells (S1 Fig), excluding the possibility that the recruitment of Rab6A and Rab33B to the LCV is an artifact in HeLa cells, which are not the preferred target cells for *L*. *pneumophila* infection. The recruitment of these Rabs to the LCV was not seen on the vacuole containing a *L*. *pneumophila ΔdotA* mutant, which is unable to secrete effectors, implying that *L*. *pneumophila* manipulates these Rabs using its effectors.

Next, we examined whether Rab6A and Rab33B are required for the intracellular pathogenesis of *L*. *pneumophila*. To test this, we performed siRNA knockdown of Rab6A and Rab6A’ (hereafter collectively called Rab6A), Rab33B, and, as a control, Rab7. These siRNAs successfully suppressed the expression of each Rab, and loss of Rab6A as well as Rab33B but not Rab7 markedly blocked intracellular growth of *L*. *pneumophila* (Fig 1C). Furthermore, the GDP-locked inactive (T to N substitution) forms of Rab6A and Rab33B, but not GTP-locked active (Q to L substitution) ones, suppressed *Legionella* growth (S2A and S2B Fig), suggesting that *L*. *pneumophila* manipulates Rab6A and Rab33B for its replication. Of note, the inactive forms of Rab6A (S2C Fig) and Rab33B (S2D Fig) were preferentially recruited to the LCV, as in the case of Rab1 recruitment by the GEF, DrrA [11]. This may reflect the fact that GEFs exhibit higher affinities for the GDP-locked form of Rabs than the GTP-locked form.

To further assess Rab6A and Rab33B manipulation by *L*. *pneumophila*, we monitored LCV maturation in the presence or absence of Rab6A and Rab33B using two protein markers, Sec22b (an ERDV-contained SNARE protein) and Sec61β (a rough ER localization protein): the former and the latter can monitor the progression of remodeling events, including the recruitment of ERDVs to the LCV, and the association of the LCV with the ER, respectively. As shown in Fig 1D, the efficient recruitment of Sec22b-containing ERDVs to the LCV was observed at 1 h post infection in Rab6A- and Rab33B-silenced cells, similar to mock-treated cells, implying that loss of both Rab proteins does not affect the uptake of *L*. *pneumophila* or the remodeling of the LCV. As reported for the ER proteins calnexin and glucose 6-phosphatase [4,28], the number of Sec61β-positive LCVs increased over the course of infection, and around 70% of the LCVs was detected as Sec61β-positive vacuoles at 6 h post infection (Fig 1E). In contrast to Sec22b, in Rab6A- and Rab33B-silenced cells, the ratio of Sec61β-positive LCVs was markedly lower at all time points examined (Fig 1E), suggesting that the functions of both Rab6A and Rab33B are required for the attachment of LCV with the ER after LCV remodeling.

### Rab33B on the LCV functions upstream of Rab6A

**Fig 2:**
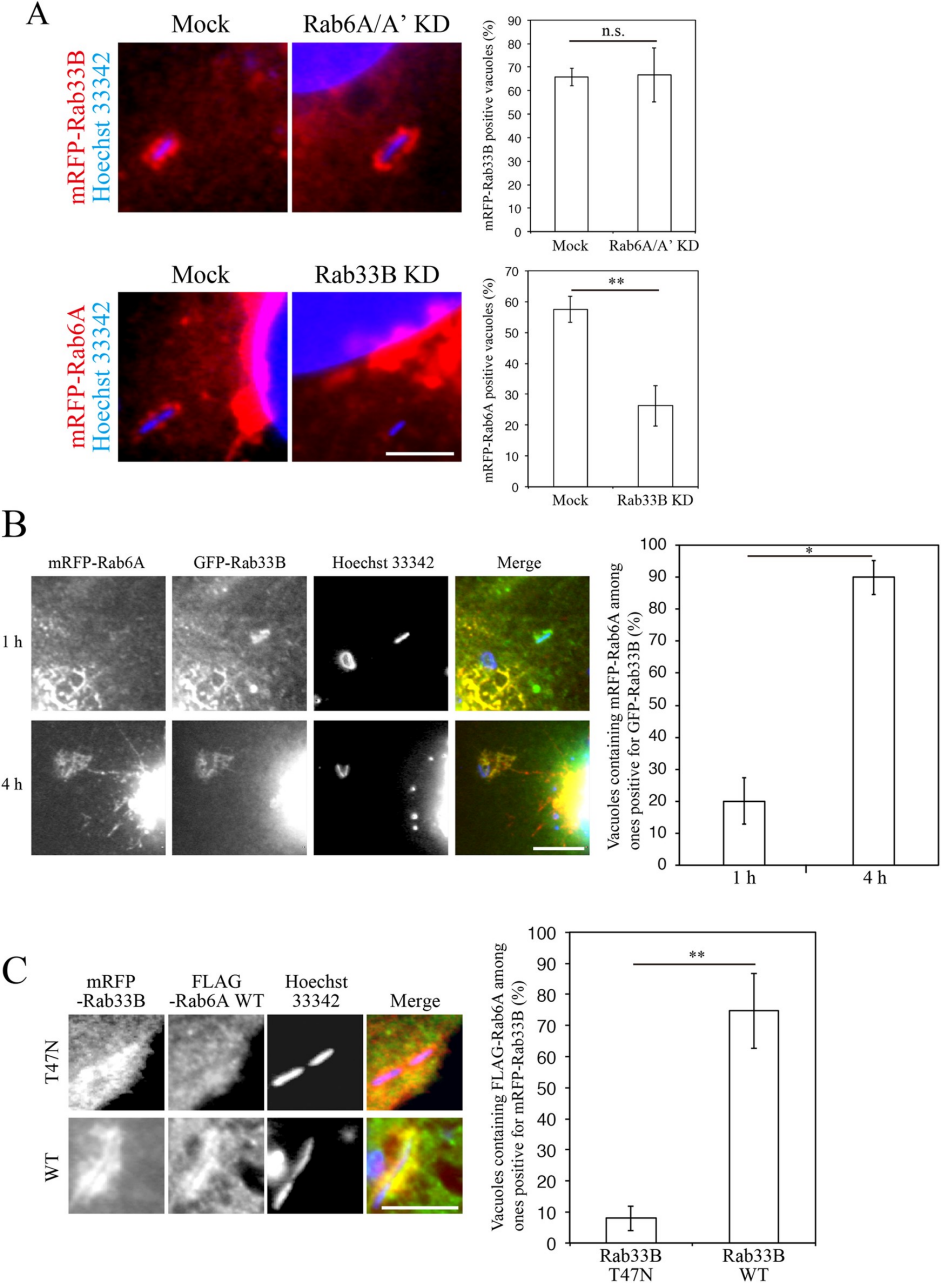
*L*. *pneumophila* modulates a Rab33B-Rab6A cascade on the LCV. (A) HeLa-FcγRII cells were transfected without (mock) or with siRNA targeting Rab33B or Rab6A/A’. At 48 h after transfection, mock and Rab6A-silenced cells and mock and Rab33B-silenced cells were additionally transfected with a plasmid encoding mRFP-Rab33B and -Rab6A, respectively, for 24 h. After transfection, the cells were infected with *L*. *pneumophila* for 4 h, fixed, and stained with Hoechst 33342. Bar, 5 μm. The graphs show the percentage of vacuoles positive for mRFP-Rab33B (top) and mRFP-Rab6A (bottom). Values are the mean ± SD (*n* = 3, 100 vacuoles were scored in each experiment). n.s., not significant. **P < 0.01 (Student’s *t* test). (B) HeLa-FcγRII cells were co-transfected with plasmids for mRFP-Rab6A and GFP-Rab33B for 24 h. After transfection, the cells were infected with *L*. *pneumophila* for 1 h (top row) or 4 h (bottom row), fixed, and stained with Hoechst 33342. Bar, 5 μm. The graph shows the percentage of vacuoles containing mRFP-Rab6A in the vacuoles positive for GFP-Rab33B. Vales are the mean ± SD (*n* = 3, 50 vacuoles were scored in each experiment). *P < 0.05 (Student’s *t* test). (C) HeLa-FcγRII cells were co-transfected with plasmids for FLAG-Rab6A and mRFP-Rab33B (T47N) (top row) or mRFP-Rab33B wild-type (bottom row) for 24 h. After transfection, the cells were infected with *L*. *pneumophila* for 4 h, fixed, and stained with an anti-FLAG antibody and Hoechst 33342. Bar, 5 μm. The graph shows the percentage of vacuoles containing FLAG-Rab6A in the vacuoles positive for mRFP-Rab33B. Vales are the mean ± SD (*n* = 3, 100 vacuoles were scored in each experiment). **P < 0.01 (Student’s *t* test).

Having established that both Rab6A and Rab33B are implicated in the association of the LCV with the ER, we next asked whether these Rabs on the LCV function independently or cooperatively. To this end, we assessed the efficiency of the recruitment of Rab33B to the LCV in the presence or absence of Rab6A, and vice versa. Silencing of Rab6A did not significantly affect Rab33B recruitment to the LCV at 4 h post infection (Fig 2A, top row). In contrast, the recruitment of Rab6A to the LCV was markedly decreased in cells silenced for Rab33B (Fig 2A, bottom row), raising the possibility that Rab33B functions upstream of Rab6A on the LCV. This is consistent with the result of the localization experiment that showed the recruitment of Rab33B to the LCV precedes that of Rab6A (Fig 2B). Furthermore, the recruitment of Rab6A was abolished on the LCV decorated with the inactive (T47N) but not wild-type Rab33B (Fig 2C), implying that activity of Rab33B on the LCV is necessary for the subsequent recruitment of Rab6A and that *L*. *pneumophila* facilitates the association of the LCV with the ER by manipulating the Rab33B-Rab6A cascade on the LCV. To confirm that Rab33B acts upstream of Rab6A and not synergistically with Rab6A, we examined the association of the LCV with the ER in cells silenced for Rab6A and Rab33B individually or simultaneously. As shown in S3 Fig, no additive effect was observed in cells silenced for both Rab6A and Rab33B, supporting the notion that Rab6A and Rab33B do not function synergistically.

Pfeffer and colleagues previously demonstrated that, the Rgp1-Ric1 complex, a Rab33B effector and Rab6 GEF, regulates Golgi distribution of Rab6A through interaction with the GTP form of Rab33B [29]. Therefore, it is possible that this complex is involved in the recruitment of Rab6A to the LCV as a Rab6A GEF. To test this, we examined whether Rgp1 and Ric1 are recruited to the LCV. As shown in S4A Fig, neither Rgp1 nor Ric1 was detected on LCVs positive for Rab33B. Furthermore, loss of Rgp1, which was shown to cause dissociation of Rab6A from the Golgi and inhibit the transport of the mannose 6-phosphate receptor from the endosome to the TGN [29], did not substantially affect the recruitment of Rab6A, as well as Rab33B, to the LCV (S4B and S4C Fig), implying that the Rab33B-meiated Rab6A recruitment to the LCV occurs in a manner independent of the Rgp1-Ric1 function.

### Recruitment of Rab33B to the LCV requires phosphoribosyl-ubiquitination of Rab33B by *Legionella* SidE family proteins

**Fig 3:**
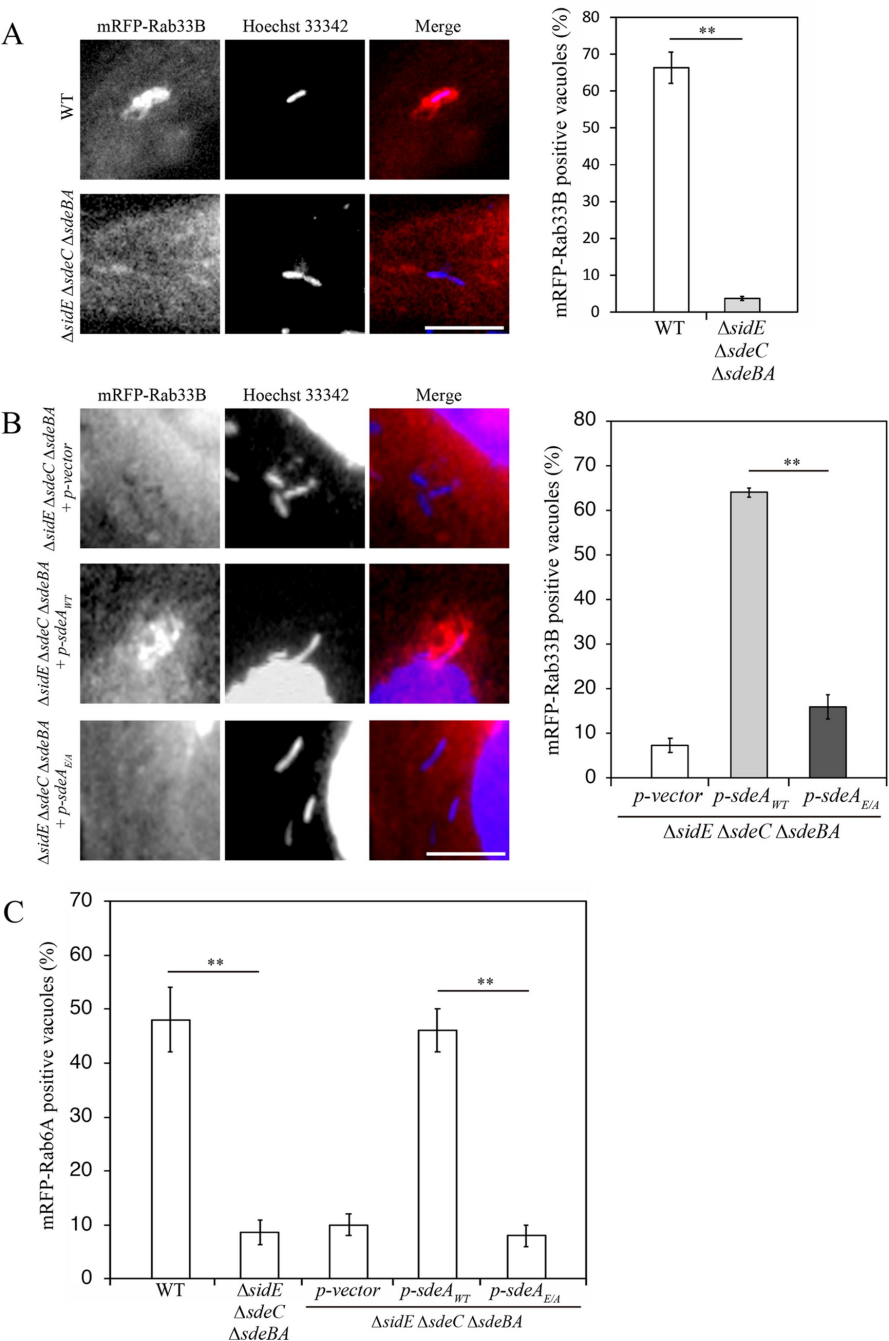
Phosphoribosyl-ubiquitination is necessary for LCV localization of Rab33B. (A) HeLa-FcγRII cells transfected with a plasmid for mRFP-Rab33B for 24 h were infected with wild-type *L*. *pneumophila* (top row) or a *ΔsidE ΔsdeC ΔsdeBA* mutant (bottom row) for 4 h. After infection, the cells were fixed and stained with Hoechst 33342. Bar, 5 μm. The graph shows the percentage of vacuoles positive for mRFP-Rab33B. Values are the mean ± SD (*n* = 3, 100 vacuoles were scored in each experiment). **P < 0.01 (Student’s *t* test). (B) HeLa-FcγRII cells transfected with a plasmid for mRFP-Rab33B for 24 h were infected with a *ΔsidE ΔsdeC ΔsdeBA L*. *pneumophila* mutant complemented with a vector (top row), SdeA wild-type (middle row), or SdeA (2EA) (bottom row) for 4 h. After infection, the cells were fixed and stained with Hoechst 33342. Bar, 5 μm. The graph shows the percentage of vacuoles positive for mRFP-Rab33B. Values are the mean ± SD (*n* = 3, 100 vacuoles were scored in each experiment). **P < 0.01 (Tukey’s test). (C) HeLa-FcγRII cells transfected with a plasmid for mRFP-Rab6A for 24 h were infected with wild-type *L*. *pneumophila*, *ΔsidE ΔsdeC ΔsdeBA L*. *pneumophila* mutant, or *ΔsidE ΔsdeC ΔsdeBA* mutant complemented with a vector, SdeA wild-type, or SdeA (2EA) for 4 h. After infection, the cells were fixed and stained with Hoechst 33342. Bar, 5 μm. The graph shows the percentage of vacuoles positive for mRFP-Rab6A. Values are the mean ± SD (*n* = 3, 50 vacuoles were scored in each experiment). **P < 0.01 (Tukey’s test).

Recent studies demonstrated that Rab33B is phosphoribosyl-ubiquitinated by the *L*. *pneumophila* SidE family of effector proteins [20,30–32], but its significance in infection as well as the major site of phosphoribosyl-ubiquitination in Rab33B remains unknown. We speculated that this modification is implicated in the function of Rab33B for *L*. *pneumophila* infection. To examine this, we analyzed the recruitment of Rab33B to the vacuole containing mutant *L*. *pneumophila*, which was depleted of all genes encoding SidE family effectors, including SidE, SdeA, SdeB, and SdeC. As shown in Fig 3A, recruitment of Rab33B to the LCV was markedly abolished in cells infected with the *L*. *pneumophila ΔsidE ΔsdeC ΔsdeBA* mutant. Importantly, Rab33B recruitment to the LCV was observed when the *ΔsidE ΔsdeC ΔsdeBA* mutant strain was complemented with wild-type *sdeA* but not a ubiquitination deficient mutant (*sdeA*_*E/A*_: substitution of E860 and E862 to A); [20]) (Fig 3B). Moreover, similar results were obtained in Rab6A recruitment to the LCV (Fig 3C), suggesting that phosphoribosyl-ubiquitination of Rab33B is necessary for its localization to the LCV.

### *L*. *Pneumophila* Promotes the Association of Rab6A with ER-resident SNARE proteins for Coalescence of the LCV with the ER

**Fig 4:**
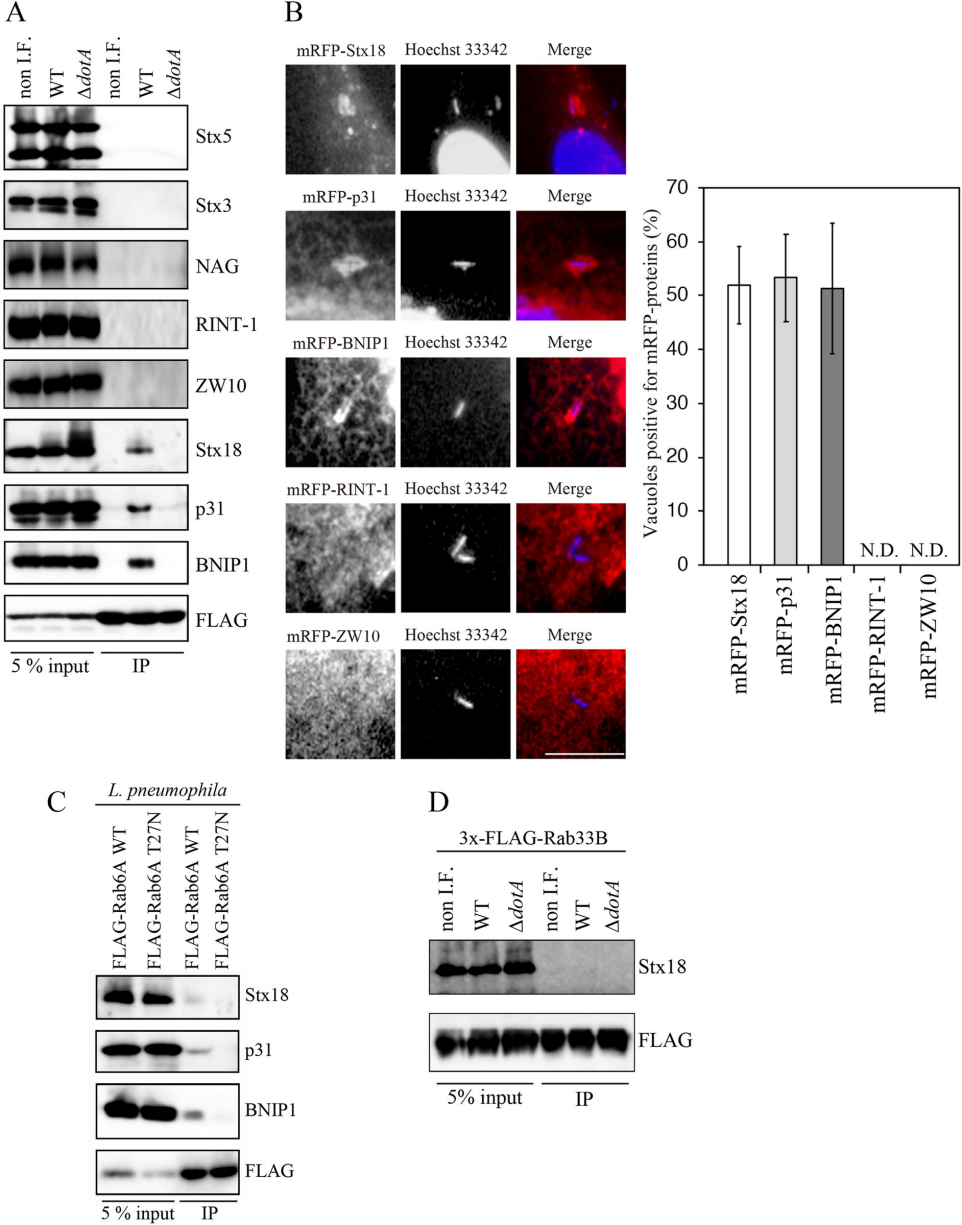
*L*. *pneumophila* enhances the association of Rab6A with ER-resident SNARE proteins. (A) HEK 293-FcγRII cells were transfected with a plasmid for 3x-FLAG-Rab6A. At 24 h after transfection, the cells were infected without or with wild-type *L*. *pneumophila* or *ΔdotA* strain at MOI 100 for 4 h. After infection, cell lysates were prepared and immunoprecipitated using anti-FLAG M2 beads. The lysates (5%) and the precipitated proteins were analyzed by the indicated antibodies. non I.F.; not infected with *L*. *pneumophila*. (B) HeLa-FcγRII cells were transfected with plasmids encoding the indicated proteins for 24 h. After transfection, the cells were infected with wild-type *L*. *pneumophila* for 4 h, fixed, and stained with Hoechst 33342. Bar, 5 μm. The graphs show the percentage of vacuoles positive for mRFP-ER-resident SNAREs. Values are the mean ± SD (n = 3, 50 vacuoles were scored in each experiment). N.D., not detected. (C) HEK 293-FcγRII cells were transfected with a plasmid encoding 3x-FLAG-Rab6A wild-type or -Rab6A (T27N). At 24 h after transfection, the cells were infected with wild-type *L*. *pneumophila* at MOI 100 for 4 h. After infection, cell lysates were prepared and immunoprecipitated using anti-FLAG M2 beads. The lysates (5%) and the precipitated proteins were analyzed by the indicated antibodies. (D) HEK 293-FcγRII cells were transfected with a plasmid for 3x-FLAG-Rab33B. Infection and immunoprecipitation were conducted as described in (A).

Rab proteins have been implicated in promoting the tethering of transport vesicles with the target membranes, and Rab-mediated tethering enhances membrane fusion in concert with SNARE proteins [33]. We therefore examined whether LCV-recruited Rab6A acts in concert with ER-resident SNARE proteins, such as Stx18, p31, BNIP1 and accessory tethering factors, neuroblastoma-amplified gene (NAG), RINT-1, and ZW10, collectively called the NRZ complex [19,34–39]. To test this, we performed immunoprecipitation using lysates from cells expressing 3x-FLAG-Rab6A which had been non-infected or infected with wild-type or *ΔdotA L*. *pneumophila*. Co-precipitation of Stx5 (ERGIC/Golgi SNARE) or Stx3 (plasma membrane SNARE) with 3x-FLAG-Rab6A was not detected in the immunoprecipitates regardless of infection with *L*. *pneumophila* (Fig 4A). Strikingly, infection of wild-type but not *ΔdotA L*. *pneumophila* promoted the association of 3x-FLAG-Rab6A with Stx18, p31, and BNIP1 (Fig 4A). Nevertheless, non of the components of the NRZ complex co-precipitated with 3x-FLAG-Rab6A even if cells were infected with wild-type *L*. *pneumophila* (Fig 4A). Consistent with this, Rab6A-associated proteins, Stx18, p31 and BNIP1, but not non-associated proteins, RINT-1 and ZW10, were detected on the wild-type LCV (Fig 4B), and we also confirmed that the accumulation of Stx18, p31 and BNIP1 to the LCV was markedly reduced in cells silenced for Rab6A (S5 Fig), suggesting that *L*. *pneumophila* enhances the Rab6A-SNARE interaction. Because *L*. *pneumophila* specifically recruits the GDP-bound form of Rab6A to the LCV (S2C Fig), we next investigated whether the *L*. *pneumophila-*facilitated association of Rab6A with the ER-resident SNAREs requires the activation of Rab6A. We conducted immunoprecipitation experiments using lysates from cells expressing 3x-FLAG-Rab6A (wild-type) or -Rab6A (T27N) after infection with wild-type *L*. *pneumophila*. Importantly, the association of Rab6A (T27N) with these proteins was not detected (Fig 4C), even though this Rab6A inactive mutant was predominantly recruited to the LCV (S2C Fig). This suggests that the activation of Rab6A by exchange of GDP for GTP is required for the interaction with these ER-resident SNAREs. Of note, co-precipitation of Stx18 with 3x-FLAG-Rab33B in wild-type *L*. *pneumophila*-infected cell lysates was not detected (Fig 4D), indicating that the ER-resident SNAREs are specific partners of Rab6A upon *L*. *pneumophila* infection.

**Fig 5:**
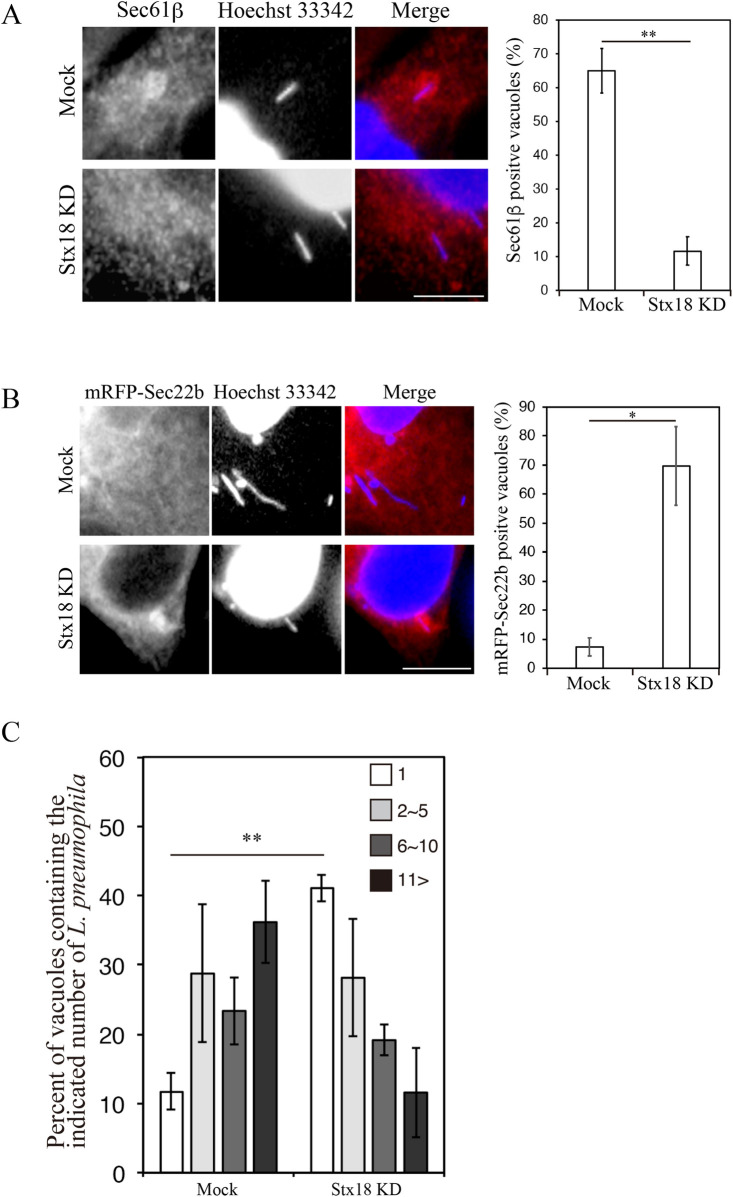
Stx18 is required for the LCV-ER association/fusion and intracellular *Legionella* growth. (A) HeLa-FcγRII cells were transfected without (mock, top row) or with siRNA targeting Stx18 (bottom row). At 48 h after transfection, the cells were infected with *L*. *pneumophila* for 4 h, fixed, and stained with an anti-Sec61β antibody and Hoechst 33342. Bar, 5 μm. The graph shows the percentage of vacuoles positive for Sec61β. Vales are the mean ± SD (*n* = 3, 100 vacuoles were scored in each experiment). **P < 0.01 (Student’s *t* test). (B) HeLa-FcγRII cells were transfected without (mock, top row) or with siRNA targeting Stx18 (bottom row). At 24 h after transfection, the cells were additionally transfected with a plasmid for mRFP-Sec22b for 24 h, infected with *L*. *pneumophila* for 6 h, fixed, and stained with Hoechst 33342. Bar, 5 μm. The graph shows the percentage of vacuoles positive for mRFP-Sec22b. Values are the mean ± SD (*n* = 3, 100 vacuoles were scored in each experiment). *P < 0.05 (Student’s *t* test). (C) HeLa-FcγRII cells were transfected without (mock) or with siRNA targeting Stx18. At 48 h after transfection, the cells were infected with *L*. *pneumophila* for 8 h at MOI 10. Intracellular growth of *L*. *pneumophila* was scored by counting bacteria residing in a single vacuole. The graph shows that percentage of vacuoles containing 1 bacterium (white bars), 2–5 bacteria (light gray bars), 6–10 bacteria (dark gray bars), and >11 bacteria (black bars). Values are the mean ± SD (*n* = 3, 100 vacuoles were scored in each experiment). *P < 0.05 (Student’s *t* test).

We next examined the effect of loss of Stx18 on the association of the LCV with the ER. Because a previous report showed that silencing of Stx18 for 72 h drastically affects the ER structure, leading to the segregation of the smooth and rough ER [40], we first searched for conditions in which Stx18 silencing does not significantly affect the ER structure. Upon 48 h silencing, the protein level of Stx18 was markedly reduced without affecting the ER morphology (S6A and S6B Fig). Therefore, we employed 48-h silencing in infection experiments. As shown in Fig 5A, at 4 h post infection, the time in which the LCV is supposed to associate with the ER, the number of Sec61β-positive LCVs was significantly lower in cells silenced for Stx18 than in mock-treated cells. On the other hand, a robust signal of mRFP-Sec22b recruited to the LCV during the remodeling stage was still detectable on the LCV in Stx18-silenced cells at 6 h post infection even though its signal disappeared around the LCV in mock-treated cells, likely due to diffusion of mRFP-Sec22b into the ER after ER-LCV fusion (Fig 5B). These results suggest that the fusion of the LCV with the ER membrane is blocked by silencing of Stx18. Consistently, loss of Stx18 blocked intracellular growth of *L*. *pneumophila* (Fig 5C).

## Discussion

**Fig 6:**
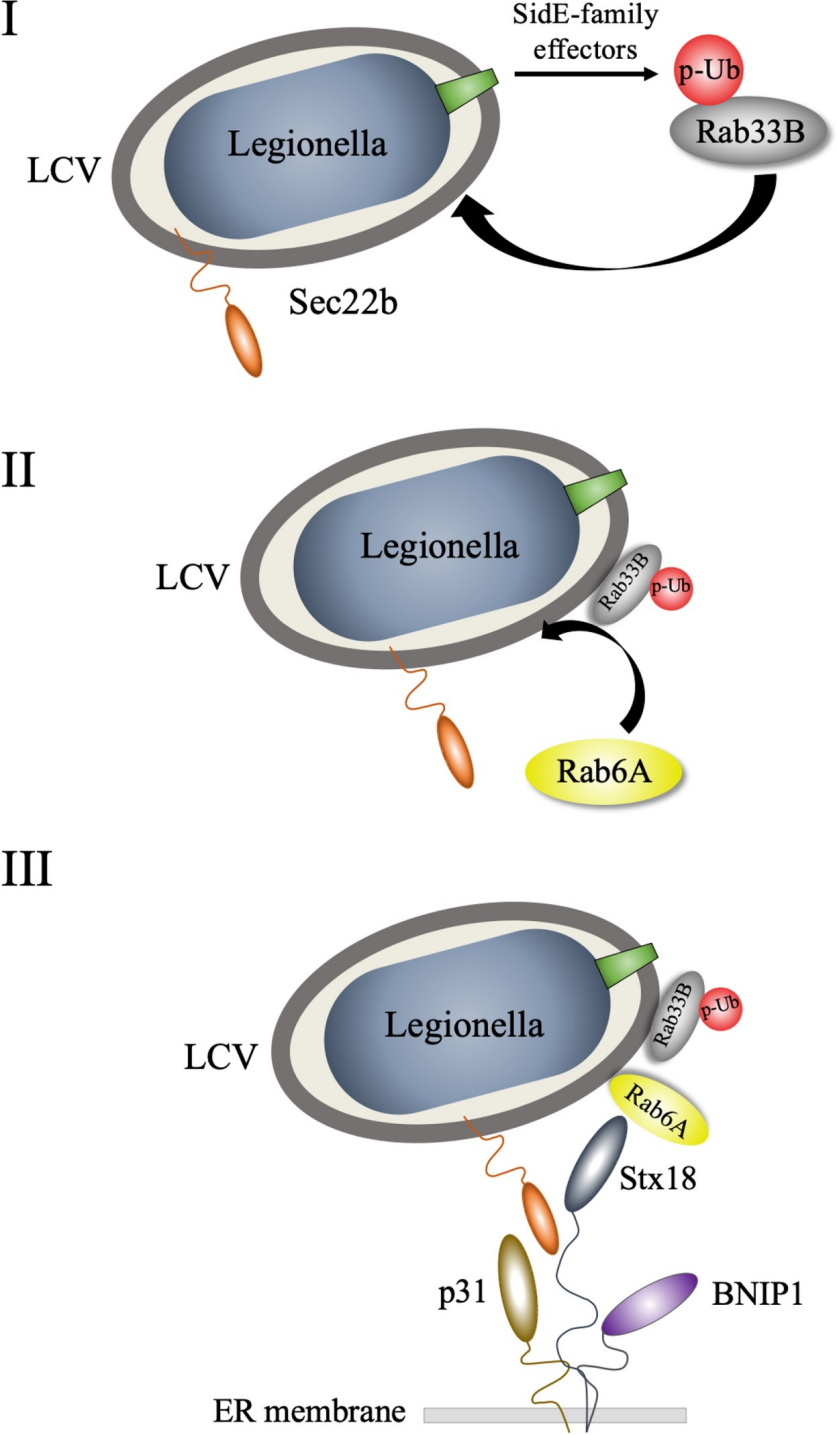
Working model for LCV association with the ER after remodeling. For details, see the text.

Based on our present results, we suggest a working model for LCV association with the ER after remodeling (Fig 6). Rab33B is phosphoribosyl-ubiquitinated by SidE family effectors, and this modification facilitates the recruitment of Rab33B to the LCV (I). And then, the LCV-attached Rab33B recruits Rab6A to the LCV (II). After this, Rab6A associates with ER-resident SNAREs to promote the efficient membrane fusion of the LCV with the ER (III).

The present observations shed light on the physiological significance of phosphoribosyl ubiquitination of Rab33B: this ubiquitination is a signal for LCV distribution of Rab33B. Because of the lack of compelling evidence for the involvement of Rab33B in *L*. *pneumophila* infection, the physiological significance of this unique modification has been obscure. In such a situation, our results unequivocally demonstrate the function of Rab33B on the LCV, i.e., the recruitment of Rab6A to the LCV, is necessary for the association of the LCV with the ER. Consistent with this, Dikic and colleagues showed the perturbation of the LCV-ER association in vacuole containing an *L*. *pneumophila* strain lacking SidE family effectors [41].

We show that phosphoribosyl ubiquitination is required for the recruitment of Rab33B to the LCV, but our preliminary data imply that other effector(s) is required for the recruitment of phosphoribosyl ubiquitinated Rab33B to the LCV because Rab33B was not present on the vacuole containing an isogenic *Legionella* strain lacking five genomic fragments (a pentuple mutant; [42]) which retains all genes encoding SidE family proteins (S7 Fig).

In the case of Rab1 recruitment to the LCV, DrrA interacts with Rab1-GDP, and the GDP-form of Rab1 is specifically recruited to the LCV [11,12]. We observed a similar nucleotide dependency for Rab33B distribution on the LCV (S2D Fig). These results suggest that *L*. *pneumophila* possess effector(s) that plays a critical role in the recruitment of the phosphoribosyl ubiquitinated Rab33B-GDP to the LCV and the exchange of nucleotide for Rab33B. Consistent with this idea, the GDP-form of Rab33B is also targeted by SidE family proteins [20]. Identification of the effector(s) is currently in progress in our laboratory. Because the host Rab33B GEF remains unidentified, the identification and structural analysis of the *Legionella* GEF for Rab33B may provide a clue for the identification of the host Rab33B GEF.

Moreover, identification of the factor(s) mediating the Rab33B-Rab6A cascade on the LCV is also important. Although the Rgp1-Ric1 complex has been shown to act as an effector for Rab33B and a GEF for Rab6A in the host [29], neither protein was recruited to the LCV, and loss of Rgp1 did not affect the integrity of the Rab33B-Rab6A cascade on the LCV, suggesting that unknown protein(s) mediates the Rab33B-Rab6A cascade on the LCV. Identification of the factor responsible for the Rab33B-Rab6A cascade may provide a novel mechanistic insight into Rab6A/Rab33B-mediated Golgi-to-ER trafficking.

It is also worth mentioning that the NRZ complex does not participate in the association of Rab6A with ER-resident SNAREs and recruited to the LCV. It seems that *L*. *pneumophila* utilizes certain, but not all, components of the host complex. In the case of the exocyst complex that comprises 8 subunits and functions in the tethering of exocytic vesicles with the plasma membrane, only Sec5, Sec6, and Sec15 are required for DrrA-mediated ERDV recruitment to the plasma membrane-derived LCV [16]. *L*. *pneumophila* may secrete tethering factor(s) that supports the attachment of Rab6A-assocsitated vesicles to the ER. Of note, Rab6A can not bind or binds only weakly and below the threshold of detection by immunoprecipitation to Stx18 in the absence of *L*. *pneumophila* (Fig 4A), implying that such effector(s) efficiently assists the interaction of Rab6A with Stx18-containing ER SNAREs. Because previous work shows that *Legionella* effector LidA binds the GTP form of Rab6A’ and prevents Rab6A’ from GAP-mediated inactivation [43], examination of whether LidA is involved in the interaction between Rab6A and ER-resident SNAREs is needed.

Hilbi and colleagues demonstrated that the LCV membrane remains separate from the ER and it does not fuse with the ER in *Dictyostelium discoideum* [44], and this finding contradicts our data in which the LCV membrane fuses with the ER (Fig 5B). Although the possibility of this contradiction might be due to species (protozoa cell vs animal cell), further investigation is required.

## Materials and methods

### Antibodies

Mouse monoclonal antibodies against calnexin (610523) was purchased from BD Biosciences Pharmingen. Rabbit polyclonal antibodies against Rab6A and Rab33B were purchased from Gene-Tex (GTX110646) and Protein-tech (27349-1-AP), respectively. Rabbit polyclonal antibodies against FLAG, Sec61β, Stx3, and Rab7 were purchased from Sigma-Aldrich (F7425), Protein-tech (14846-1-AP), Abcam (ab86669), and Cell Signaling (9367), respectively. Rabbit polyclonal and goat polyclonal antibodies against RFP and RTN4 were purchased from Medical and Biological Laboratories (PM005) and Santa Cruz (sc-11027; a currently discontinued product), respectively. Rabbit polyclonal antibodies against Stx5, Stx18, p31, BNIP1, NAG, RINT-1, and ZW10 were prepared in this laboratory [34–36,39]. Rabbit polyclonal antibodies against *L*. *pneumophila* were a gift from Dr. Craig Roy at Yale University School of Medicine or prepared in our laboratory. Mouse polyclonal antibodies against *L*. *pneumophila* were prepared in our laboratory.

### Cell culture and bacterial strain

Maintenance of HeLa-FcγRII cells and HEK 293-FcγRII cells was described previously [6,16]. J774.1 macrophage cells were grown in RPMI-1640 medium supplemented with 50 IU/ml penicillin, 50 μg/ml streptomycin, and 10% fetal calf serum. Growth of *L*. *pneumophila* strains (wild-type; Lp01, Lp01 *ΔdotA* mutant; CR58) was maintained as described previously [16]. Lp01 *ΔsidE ΔsdeC ΔsdeBA* mutant was constructed by allelic exchanges as described previously [45]. Site-directed mutagenesis of *sdeA* to construct the *sdeA*_E860A E862A_ (*sdeA*_E/A_) mutant was conducted on *sdeA* cloned into pUC18. To construct the *L*. *pneumophila* expression plasmids, pMMB207-3xFLAG-*sdeA* and pMMB207-3xFLAG-*sdeA*_E/A_, the *sdeA* locus was amplified from pUC18-*sdeA* and pUC18-*sdeA*_E/A_, respectively, and cloned into the pMMB207-3xFLAG vector [46]. The conditions for the growth of *L*. *pneumophila* strains (a thymidine auxotroph mutant (Lp02) and a pentuple mutant) were described previously [42].

### Preparation of *L*. *pneumophila*-infected cells

*L*. *pneumophila* was opsonized by rabbit or mouse anti-*Legionella* antibodies. Cells were spread in density of 1x 10^6^ cells and were infected with a wild-type or a *ΔdotA* strain for the indicated MOI. At 1 h after infection, the cells were washed extensively and cultured in fresh DMEM for HEK293-FcγRII cells and fresh α-MEM for HeLa-FcγRII cells.

### Immunofluorescence microscopy

Cells were fixed with 4% paraformaldehyde/PBS for 20 min at room temperature and permeabilized with 0.2% Triton X-100 for 15 min at room temperature. In infection experiments, separation of extracellular and intracellular bacteria was performed as described previously [16]. The samples were observed under a fluorescence microscope (Olympus BX50).

### Immunoprecipitation

Lysates from cells infected with *L*. *pneumophila* for immunoprecipitation were prepared as described previously [6]. For immunoprecipitation experiments, lysates were incubated with anti-FLAG M2 agarose (Sigma-Aldrich) for 1 h at 4°C. After incubation, the beads were washed extensively, and the precipitated proteins were eluted using SDS sample buffer.

### RNA interference

Cells were transfected with siRNA using Oligofectamine (Invitrogen) according to the manufacture’s protocol. The siRNA targeting Rab6A/A’ (5’-AAGACAUCUUUGAUCACCAGA-3’), Rab33B (5’-GAUAGAAGAAUGCAAACAA-3’), Rab7 (5’-GGAUGACCUCUAGGAAGAA-3’) and Rgp1 (5’-CAGUGAUGGCCGAGGGAAA-3’) were purchased from Japan Bioservice (Asaka, Japan). The target sequence of Stx18 was described previously [40].

### Quantification and statistics

The results from each experiment were averaged and expressed as the mean with SD and analyzed by a paired Student’s *t* test (2 groups) or a Tukey’s test (more 3 groups). All the experiments were repeated at least three times.

## Supporting information

S1 Fig Recruitment of Rab6A and Rab33B to the LCV in J774.1 macrophage. J774.1 macrophages were infected with with *L*. *pneumophila* for the indicate times at MOI 25. After infection, cells were fixed and stained with an anti-Rab6A or -Rab33B antibody and Hoechst 33342. Images show that typical vacuoles positive and negative for endogenous Rab6A (top and second rows) and Rab33B (third and bottom rows). Bar, 5 μm. The graphs show the percentage of vacuoles positive for endogenous Rab proteins. Values are the mean ± SD (n = 3, 50 vacuoles were scored in each experiment).
(TIF)

S2 Fig Effect of nucleotide state of Rab6A or Rab33B on the intracellular growth of *L. pneumophila* and LCV recruitment. (A and B) HeLa-FcγRII cells were transfected with a plasmid for mRFP-Rab6A (T27N) or -Rab6A (Q72L) (A) or a plasmid for mRFP-Rab33B (T47N) or -Rab33B (Q92L) (B) for 24 h. After transfection, the cells were infected with *L*. *pneumophila* for 8 h at MOI 10. The cells were stained with an anti-*L*. *pneumophila* antibody and the number of replicated *L*. *pneumophila* in mRFP-Rab proteins expressing cells was counted. The graph shows that the average of the number of replicated *L*. *pneumophila*. Values are the mean ± SD (n = 4, 20 vacuoles were scored in each experiment). *P < 0.05, **P < 0.01 (Tukey test). (C and D) HeLa-FcγRII cells were transfected with a plasmid for mRFP-Rab6A (T27N) or -Rab6A (Q72L) (C) or plasmid for mRFP-Rab33B (T47N) or -Rab33B (Q92L) (D) for 24 h. After transfection, the cells were infected with *L*. *pneumophila* for 4 h, fixed, and stained with Hoechst 33342. Bar, 5 μm.
(TIF)

S3 Fig Effect of both Rab6A and Rab33B silencing on the association of LCV with the ER. (A) HeLa-FcγRII cells were transfected without (mock) or with siRNA targeting Rab6A/A’, Rab33B, or both Rab6A/A’ and Rab33B. At 72 h after transfection, the efficiency of Rab protein silencing was assessed by the indicated antibodies. (B) Mock and siRNA transfected HeLa-FcγRII cells were infected with *L*. *pneumophila* for 6 h, fixed, and stained with an anti-Sec61β antibody and Hoechst 33342. The graph shows the percentage of vacuoles positive for Sec61β at the indicate times. Values are the mean ± SD (*n* = 3, 50 vacuoles were examined in each experiment). **P < 0.01 (Tukey test).
(TIF)

S4 Fig Rgp1-Ric1 complex is not implicated in the Rab33B-Rab6A cascade on the LCV. (A) HeLa-FcγRII cells were co-transfected with plasmids for mRFP-Rab33B and FLAG-Rpg1 (top row) or -Ric1 (bottom row). At 24 h after transfection, the cells were infected with *L*. *pneumophila* for 4 h, fixed, and stained with an anti-FLAG antibody and Hoechst 33342. Bar, 5 μm. (B) HeLa-FcγRII cells were transfected with siRNA targeting Rgp1. At 48 h after transfection, the cells were additionally transfected with an mRFP vector or an mRFP-Rgp1 vector for 24 h and lysed, and the equal amounts of lysates were analyzed using the indicated antibodies. (C) HeLa-FcγRII cells were transfected without (mock) or with siRNA targeting Rgp1. At 48 h after transfection, the cells were additionally transfected with a plasmid for mRFP-Rab6A (top and second rows) or mRFP-Rab33B (third and bottom rows) for 24 h, infected with *L*. *pneumophila* for 4 h, fixed, and stained with Hoechst 33342. Bar, 5 μm. The graphs on the right show that percentage of vacuoles positive for mRFP-Rab6A and mRFP-Rab33B, respectively. Values are the mean ± SD (*n* = 3, 100 vacuoles were scored in each experiment). n.s.; not significant (Student’s *t* test).
(TIF)

S5 Fig The Effect of Rab6A silencing on the accumulation of ER-SNAREs to the LCV. HeLa-FcγRII cells were transfected without (left three columns) or with siRNA targeting Rab6A/A’ (right three columns). At 48 h after transfection, the cells were additionally transfected with a plasmid for mRFP-ER SNARE proteins for 24 h, infected with *L*. *pneumophila* for 4 h, fixed, and stained with Hoechst 33342. Bar, 5 μm. The graph shows the percentage of vacuoles positive for mRFP-ER SNARE proteins. Values are the mean ± SD (n = 3, 50 vacuoles are scored in each experiment). N.D., not detected. *P < 0.05 (Student’s *t* test).
(TIF)

S6 Fig Assessment of Stx18 silencing. (A) HeLa-FcγRII cells were transfected without (mock) or with siRNA targeting Stx18. At 48 h after transfection, the cells were lysed, and the equal amounts of proteins were analyzed using the indicated antibodies. (B) HeLa-FcγRII cells were transfected without (mock, top row) or with siRNA targeting Stx18 (bottom row). At 48 h after transfection, the cells were fixed and stained with the indicated antibodies. Bar, 5μm.
(TIF)

S7 Fig Distribution of Rab33B on the vacuole containing the pentuple mutant strain of *L. pneumophila*. HeLa-FcγRII cells were transfected with a plasmid for mRFP-Rab33B. At 24 h after transfection, cells were infected with Lp02 or Lp02 lacking five genomic fragments (pentuple) for 4 h, fixed and stained with Hoechst 33342. Bar, 5 μm. The graph shows the percentage of vacuoles positive for mRFP-Rab33B. Values are the mean ± SD (n = 3, 50 vacuoles were scored in each experiment). **P < 0.01 (Student’s *t* test).
(TIF)
