# Supplementary figures and images for: Legionella hijacks the host Golgi-to-ER retrograde pathway for the association of Legionella-containing vacuole with the ER
Source: PLoS Pathog. 2021 Mar 24;17(3):e1009437. doi: 10.1371/journal.ppat.1009437 (PMC8021152; doi:10.1371/journal.ppat.1009437)

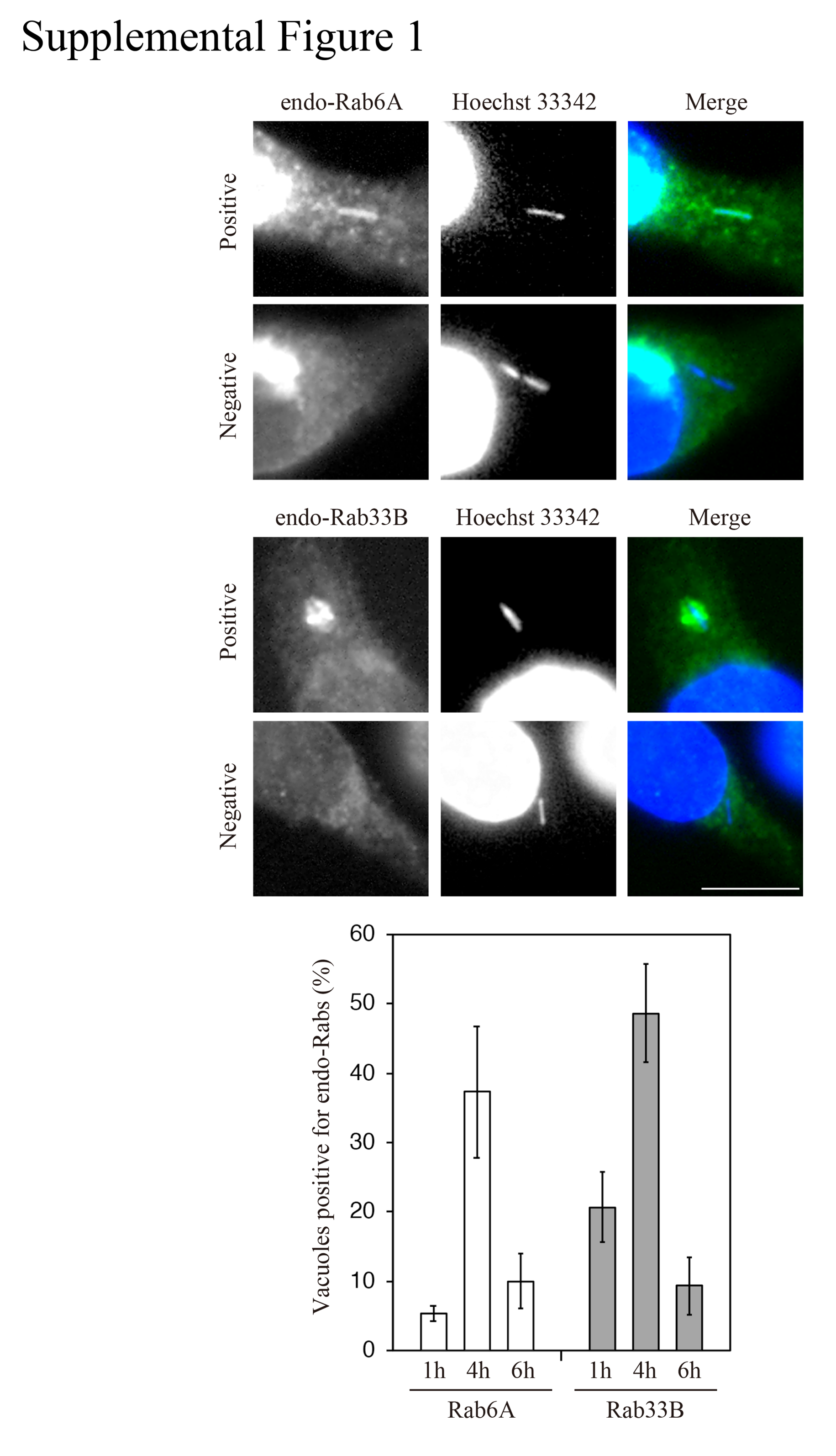

Supplement: S1 Fig — J774.1 macrophages were infected with with L. pneumophila for the indicate times at MOI 25. After infection, cells were fixed and stained with an anti-Rab6A or -Rab33B antibody and Hoechst 33342. Images show that typical vacuoles positive and negative for endogenous Rab6A (top and second rows) and Rab33B (third and bottom rows). Bar, 5 μm. The graphs show the percentage of vacuoles positive for endogenous Rab proteins. Values are the mean ± SD (n = 3, 50 vacuoles were scored in each experiment). (TIF) [file ppat.1009437.s001.tif]

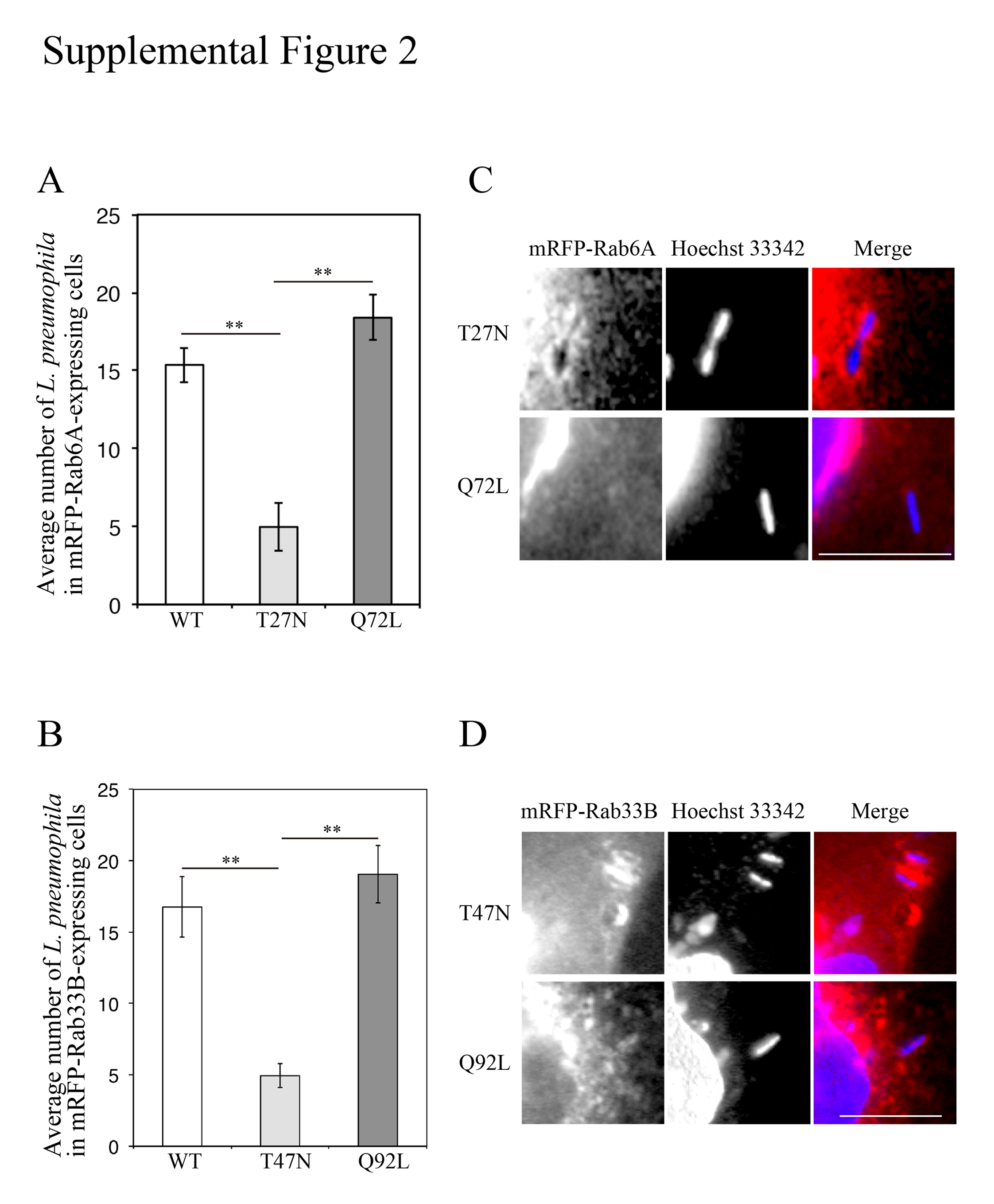

Supplement: S2 Fig — (A and B) HeLa-FcγRII cells were transfected with a plasmid for mRFP-Rab6A (T27N) or -Rab6A (Q72L) (A) or a plasmid for mRFP-Rab33B (T47N) or -Rab33B (Q92L) (B) for 24 h. After transfection, the cells were infected with L. pneumophila for 8 h at MOI 10. The cells were stained with an anti-L. pneumophila antibody and the number of replicated L. pneumophila in mRFP-Rab proteins expressing cells was counted. The graph shows that the average of the number of replicated L. pneumophila. Values are the mean ± SD (n = 4, 20 vacuoles were scored in each experiment). *P < 0.05, **P < 0.01 (Tukey test). (C and D) HeLa-FcγRII cells were transfected with a plasmid for mRFP-Rab6A (T27N) or -Rab6A (Q72L) (C) or plasmid for mRFP-Rab33B (T47N) or -Rab33B (Q92L) (D) for 24 h. After transfection, the cells were infected with L. pneumophila for 4 h, fixed, and stained with Hoechst 33342. Bar, 5 μm. (TIF) [file ppat.1009437.s002.tif]

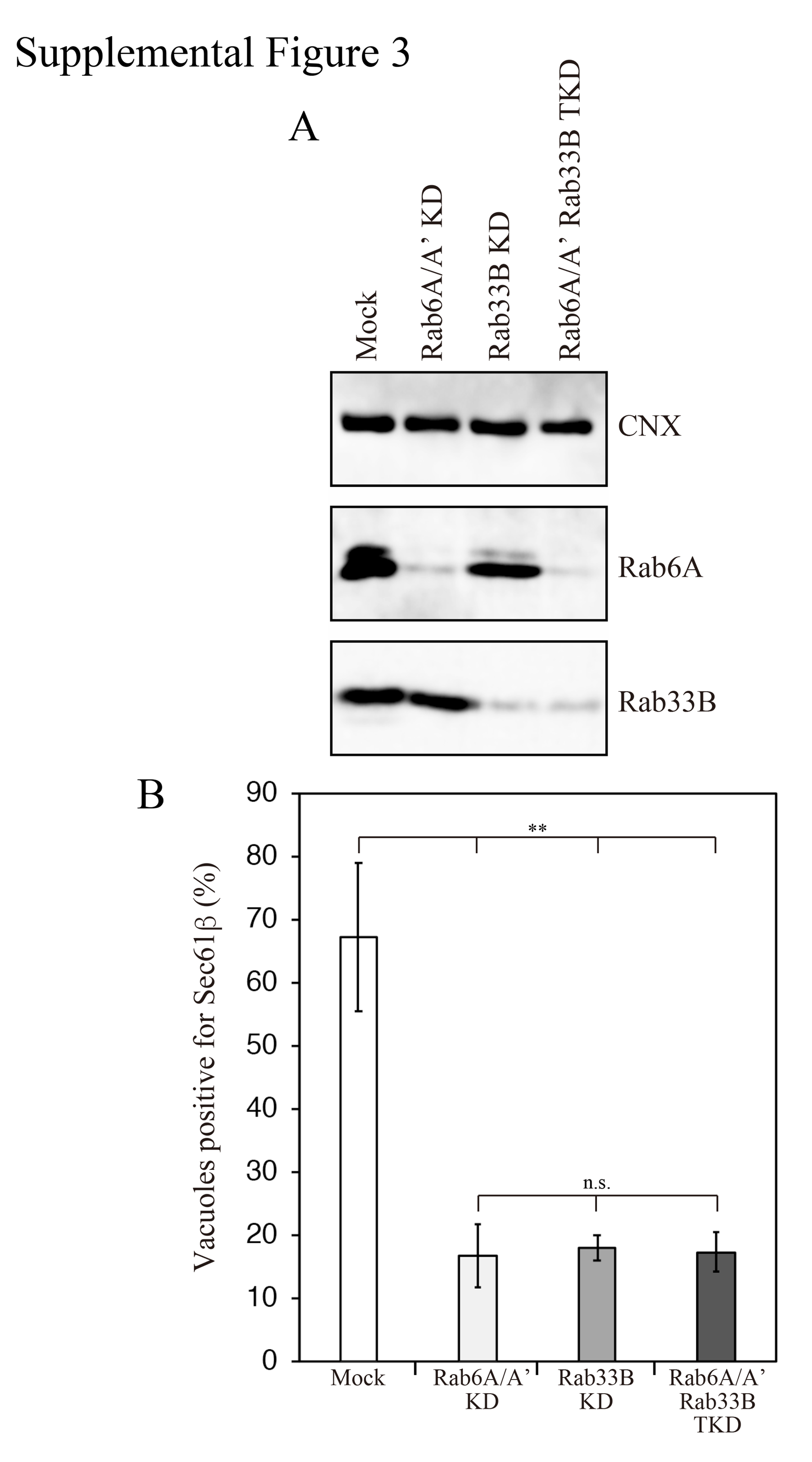

Supplement: S3 Fig — (A) HeLa-FcγRII cells were transfected without (mock) or with siRNA targeting Rab6A/A’, Rab33B, or both Rab6A/A’ and Rab33B. At 72 h after transfection, the efficiency of Rab protein silencing was assessed by the indicated antibodies. (B) Mock and siRNA transfected HeLa-FcγRII cells were infected with L. pneumophila for 6 h, fixed, and stained with an anti-Sec61β antibody and Hoechst 33342. The graph shows the percentage of vacuoles positive for Sec61β at the indicate times. Values are the mean ± SD (n = 3, 50 vacuoles were examined in each experiment). **P < 0.01 (Tukey test). (TIF) [file ppat.1009437.s003.tif]

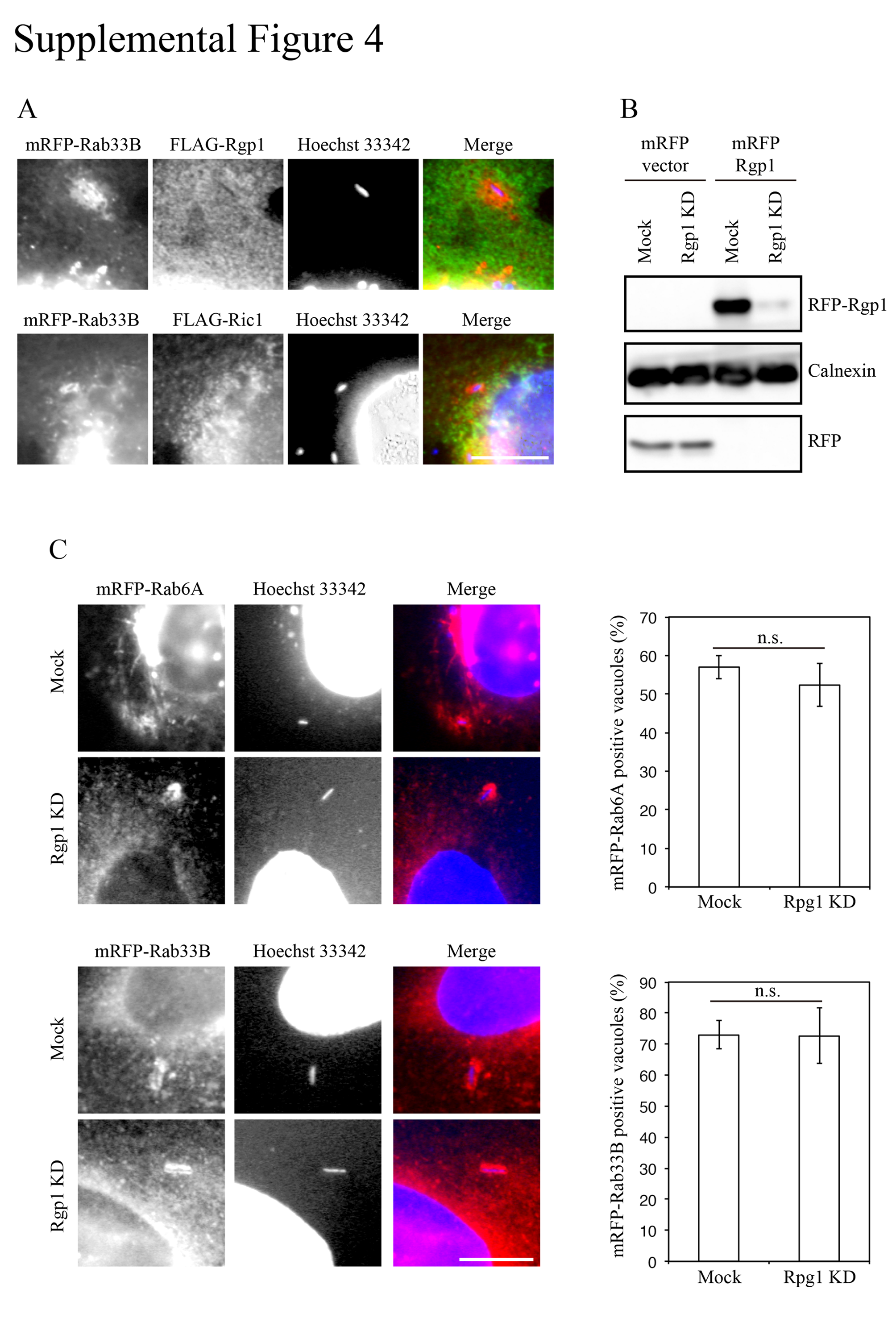

Supplement: S4 Fig — (A) HeLa-FcγRII cells were co-transfected with plasmids for mRFP-Rab33B and FLAG-Rpg1 (top row) or -Ric1 (bottom row). At 24 h after transfection, the cells were infected with L. pneumophila for 4 h, fixed, and stained with an anti-FLAG antibody and Hoechst 33342. Bar, 5 μm. (B) HeLa-FcγRII cells were transfected with siRNA targeting Rgp1. At 48 h after transfection, the cells were additionally transfected with an mRFP vector or an mRFP-Rgp1 vector for 24 h and lysed, and the equal amounts of lysates were analyzed using the indicated antibodies. (C) HeLa-FcγRII cells were transfected without (mock) or with siRNA targeting Rgp1. At 48 h after transfection, the cells were additionally transfected with a plasmid for mRFP-Rab6A (top and second rows) or mRFP-Rab33B (third and bottom rows) for 24 h, infected with L. pneumophila for 4 h, fixed, and stained with Hoechst 33342. Bar, 5 μm. The graphs on the right show that percentage of vacuoles positive for mRFP-Rab6A and mRFP-Rab33B, respectively. Values are the mean ± SD (n = 3, 100 vacuoles were scored in each experiment). n.s.; not significant (Student’s t test). (TIF) [file ppat.1009437.s004.tif]

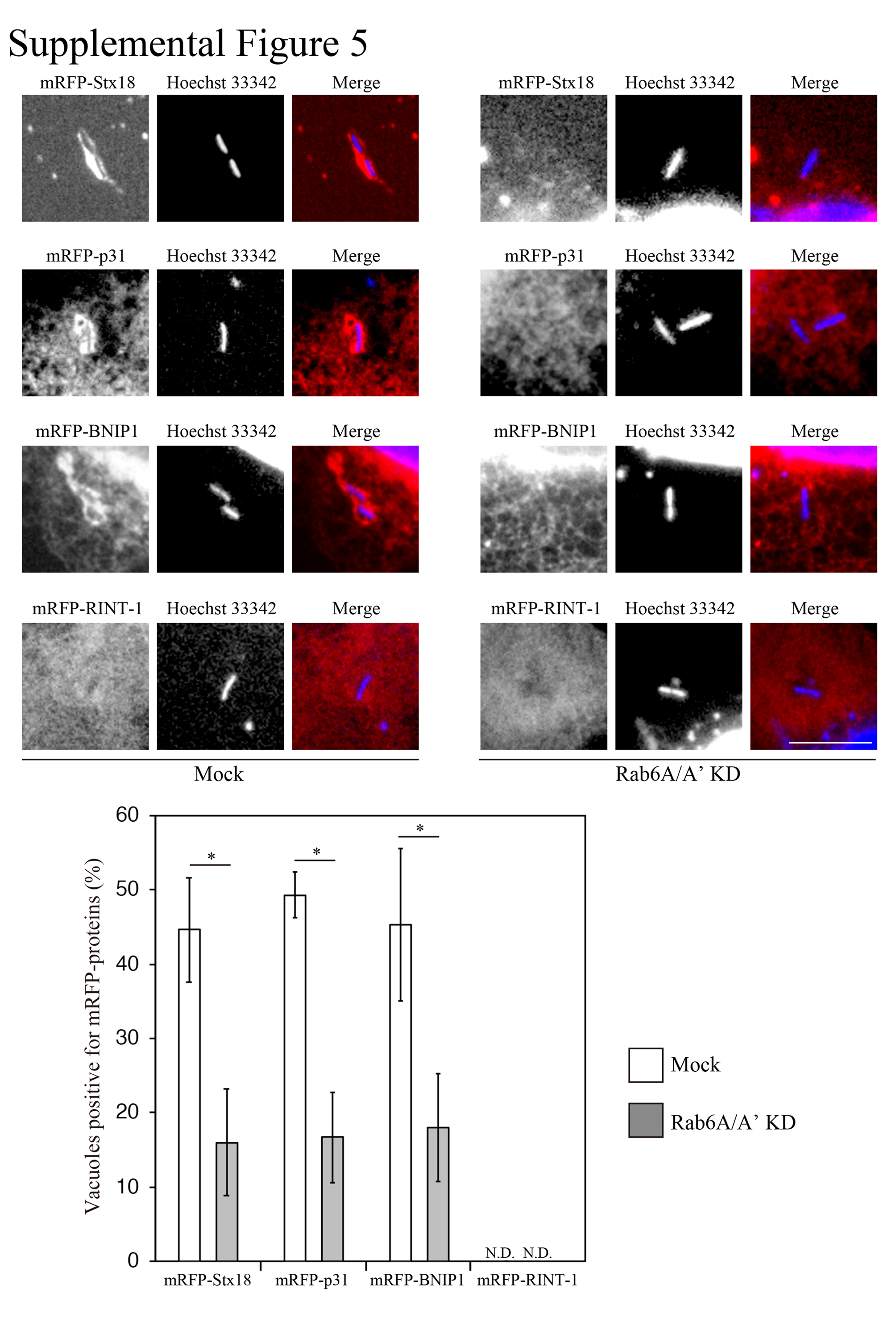

Supplement: S5 Fig — HeLa-FcγRII cells were transfected without (left three columns) or with siRNA targeting Rab6A/A’ (right three columns). At 48 h after transfection, the cells were additionally transfected with a plasmid for mRFP-ER SNARE proteins for 24 h, infected with L. pneumophila for 4 h, fixed, and stained with Hoechst 33342. Bar, 5 μm. The graph shows the percentage of vacuoles positive for mRFP-ER SNARE proteins. Values are the mean ± SD (n = 3, 50 vacuoles are scored in each experiment). N.D., not detected. *P < 0.05 (Student’s t test). (TIF) [file ppat.1009437.s005.tif]

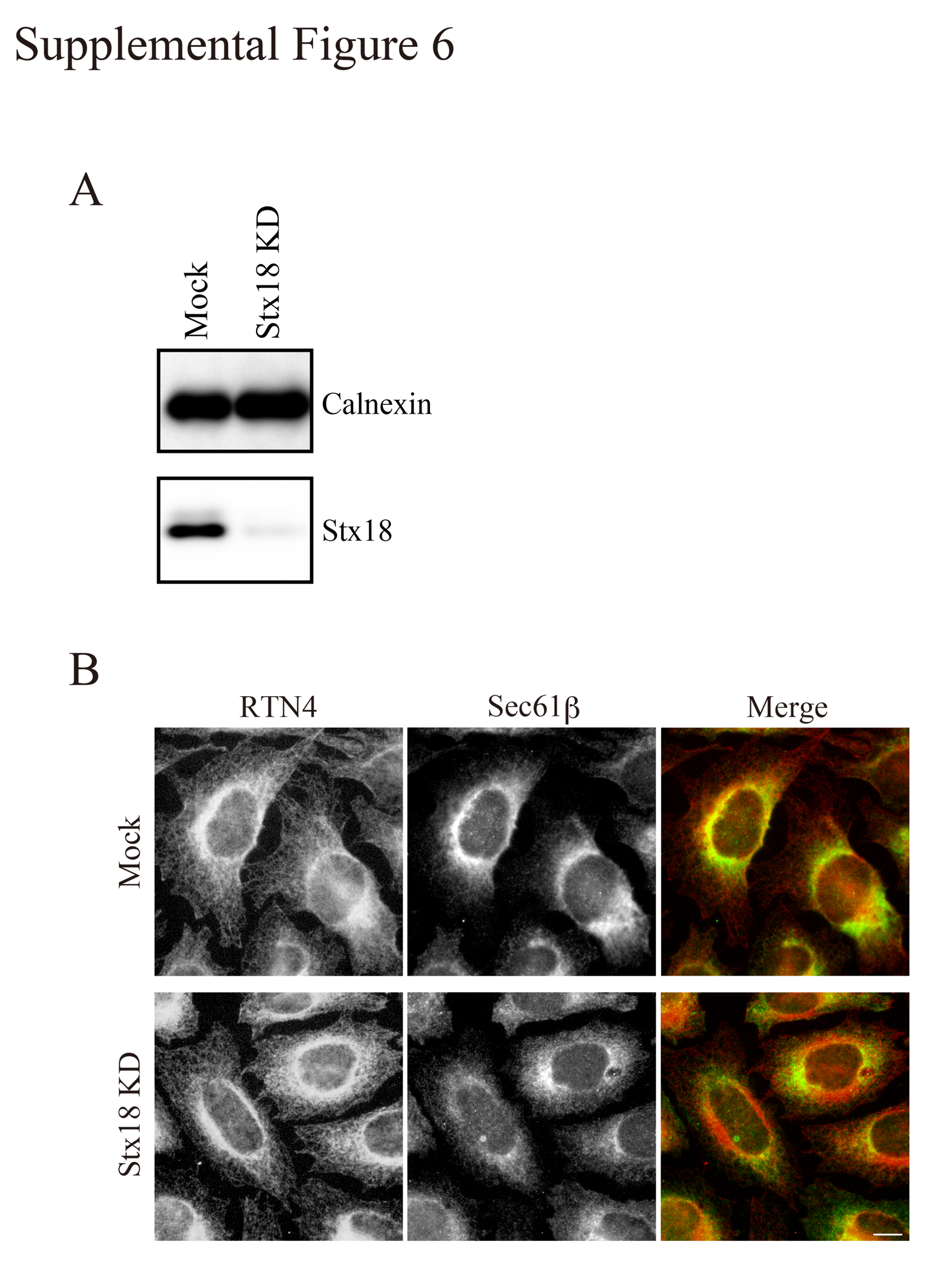

Supplement: S6 Fig — (A) HeLa-FcγRII cells were transfected without (mock) or with siRNA targeting Stx18. At 48 h after transfection, the cells were lysed, and the equal amounts of proteins were analyzed using the indicated antibodies. (B) HeLa-FcγRII cells were transfected without (mock, top row) or with siRNA targeting Stx18 (bottom row). At 48 h after transfection, the cells were fixed and stained with the indicated antibodies. Bar, 5μm. (TIF) [file ppat.1009437.s006.tif]

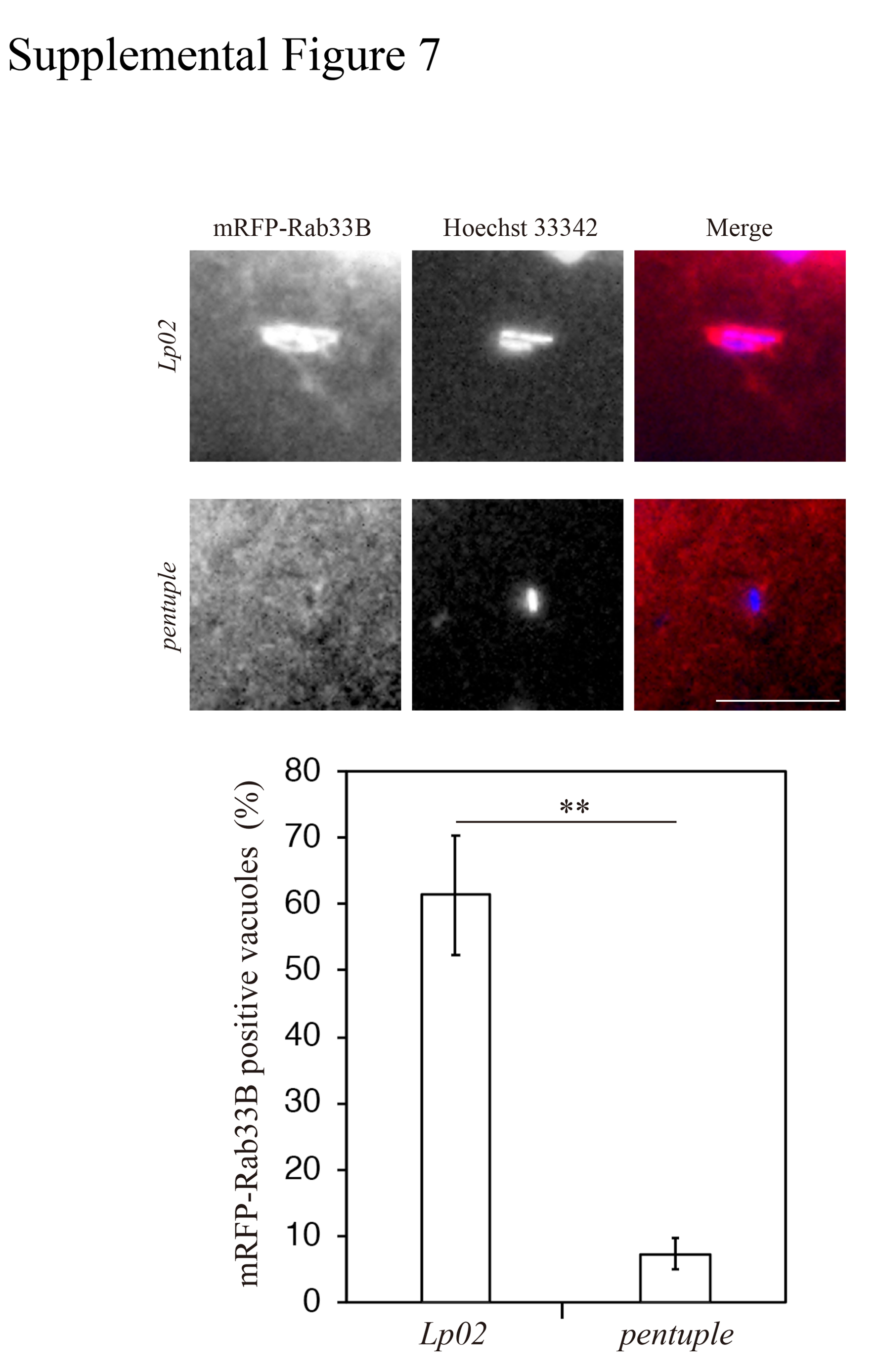

Supplement: S7 Fig — HeLa-FcγRII cells were transfected with a plasmid for mRFP-Rab33B. At 24 h after transfection, cells were infected with Lp02 or Lp02 lacking five genomic fragments (pentuple) for 4 h, fixed and stained with Hoechst 33342. Bar, 5 μm. The graph shows the percentage of vacuoles positive for mRFP-Rab33B. Values are the mean ± SD (n = 3, 50 vacuoles were scored in each experiment). **P < 0.01 (Student’s t test). (TIF) [file ppat.1009437.s007.tif]
